# Challenges in Cell Fate Acquisition to Scid-Repopulating Activity from Hemogenic Endothelium of hiPSCs Derived from AML Patients Using Forced Transcription Factor Expression

**DOI:** 10.3390/cells11121915

**Published:** 2022-06-13

**Authors:** Deanna P. Porras, Jennifer C. Reid, Borko Tanasijevic, Diana Golubeva, Allison L. Boyd, Mickie Bhatia

**Affiliations:** 1 Department of Biochemistry and Biomedical Sciences, McMaster University, Hamilton, ON L8N 3Z5, Canada; porrasd@mcmaster.ca (D.P.P.); reidjc2@mcmaster.ca (J.C.R.); tanasb@mcmaster.ca (B.T.); golubeda@mcmaster.ca (D.G.); boydal2@mcmaster.ca (A.L.B.); 2 Michael G. DeGroote School of Medicine, McMaster University, 1200 Main Street West, MDCL 5029, Hamilton, ON L8N 3Z5, Canada

**Keywords:** acute myeloid leukemia (AML), hematopoietic stem/progenitor cell (HSPC), induced pluripotent stem cell (iPSC), hemogenic endothelium (HE), transcription factor (TF), human pluripotent stem cell-derived HSPCs (hPSC-HSPCs), xenotransplantation

## Abstract

The generation of human hematopoietic stem cells (HSCs) from human pluripotent stem cells (hPSCs) represents a major goal in regenerative medicine and is believed would follow principles of early development. HSCs arise from a type of endothelial cell called a “hemogenic endothelium” (HE), and human HSCs are experimentally detected by transplantation into SCID or other immune-deficient mouse recipients, termed SCID-Repopulating Cells (SRC). Recently, SRCs were detected by forced expression of seven transcription factors (TF) (ERG, HOXA5, HOXA9, HOXA10, LCOR, RUNX1, and SPI1) in hPSC-derived HE, suggesting these factors are deficient in hPSC differentiation to HEs required to generate HSCs. Here we derived PECAM-1-, Flk-1-, and VE-cadherin-positive endothelial cells that also lack CD45 expression (PFV^CD45−^) which are solely responsible for hematopoietic output from iPSC lines reprogrammed from AML patients. Using HEs derived from AML patient iPSCs devoid of somatic leukemic aberrations, we sought to generate putative SRCs by the forced expression of 7TFs to model autologous HSC transplantation. The expression of 7TFs in hPSC-derived HE cells from an enhanced hematopoietic progenitor capacity was present in vitro, but failed to acquire SRC activity in vivo. Our findings emphasize the benefits of forced TF expression, along with the continued challenges in developing HSCs for autologous-based therapies from hPSC sources.

## 1. Introduction

Hematopoietic stem cells (HSCs) are functionally defined by their self-renewal activity and multi-lineage differentiation potential [1,2,3]. Consequently, human HSCs possess enormous therapeutic potential in the context of HSC transplantation (HSCT) [4,5,6]. To date, HSCT remains the most prevalent and efficacious cell therapy and enables the complete restoration of the hematopoietic system (myeloid, erythroid, and lymphoid lineages) in patients after hematopoietic damaging chemo- and radiation therapy, as well as for treatment of a variety of hematological disorders such as Acute myeloid leukemia (AML). AML is a clonal disorder characterized by the accumulation of immature myeloid progenitors (AML blasts) in the bone marrow (BM) and peripheral blood of patients. The overproduction of AML blasts and inability to differentiate depletes and affects production of other normal blood cells resulting in a variety of symptoms, including anemia and infection [7]. Although the induction of remission and subsequent post-remission therapies for AML are generally effective, relapse of the disease attributes to poor long-term survival rates [8]. However, the use of HSCT is known to be curative and may, in part, be due to the replacement of leukemic stem cells (LSC)s with healthy, newly transplanted HSCs [9]. This suggests that although the majority of HSCT in AML patients are allogenic and are accompanied with benefits of graft versus leukemia (GVL) [10], autologous HSCT has significant benefits and a potential impact on disease survival. An alternative source of HSCs would be directly applicable to AML disease management and several other disorders, where pluripotent stem cells (PSCs) have been noted as an ideal renewable source [11] capable of generating these highly sought-after cells.

The generation of HSCs from pluripotent sources requires an understanding of development biology involving HSC genesis in the mammal. HSCs emerge in the second and definitive wave of hematopoiesis derived from the endothelial microenvironment of the dorsal aorta within the aorta–gonad–mesonephros (AGM) region [12,13,14]. Accordingly, endothelial markers are expressed in early hematopoietic cells, underscoring the direct link of hematopoietic and endothelial cells from a common endothelial precursor [15,16]. Lineage-tracing studies provided direct evidence for a specialized hemato-endothelial precursor, that has been broadly termed a hemogenic endothelium (HE) [17,18]. HE cells migrate to the fetal liver where they expand dramatically, and then migrate to the BM at birth and sustain hematopoiesis throughout adulthood [14,19]. The developmental origins and molecular cues driving hematopoiesis and subsequent HSC formation have been investigated to recapitulate this process in vitro for HSC generation from embryonic stem cells (ESCs). Specifically, the ability to generate human induced pluripotent stem cells (hiPSCs) [20] that share phenotypic, molecular, and functional hallmarks with human ESCs, provides opportunities to mimic developmental programs in vitro through the utilization of growth factors that regulate similar signaling cascades. Unfortunately, similar to the initial pioneering attempts to generate HSCs from human PSCs, extensive investigation and more sophisticated procedures to derive transplantable HSCs from hPSC sources have been consistently unsuccessful when strictly using morphogens and cytokines [21,22,23,24,25]. This is likely due to the complex coordinated orchestration and reception of temporally and spatially dynamic signaling pathways (NOTCH, FGF (fibroblast growth factor), EGF (epidermal growth factor), Wingless/WNT, HEDGEHOG, BMP/TGF, HIPPO, cytokine/JAK/STATs, TNF/IFN/NFB, JNK, and RAR) crucial to hematopoietic development [26].

HSCs are operationally defined based on their ability to give rise to the reconstitution (self-renewal) of all blood lineages (multi-lineage) following transplantation into patients, clinically speaking, and immunodeficient mice, experimentally. Currently, the severe combined immunodeficiency (SCID)-repopulating cell (SRC) assay has been considered a gold standard for the surrogate assessment of human HSC activity [27,28]. The generation of abundant alternative sources of HSCs remain an elusive, but critical need for expanded HSC applications. In the context of AML, the generation of AML patient-specific HSCs that are devoid of the leukemic aberration(s) that affect the patient’s hematopoietic tissue would provide a transformative approach in establishing a healthy autologous source during the management of AML patients [29]. Robust long-term engraftment of hPSC-derived SRCs remains a major goal to the clinical and scientific communities alike [25,30]. Recently, two landmark studies recapitulated the endothelial-to-hematopoietic lineage transition (EHT) to generate putative mouse HSCs [31] or human SRCs [32] from the Rafii and Daley groups, respectively. In the case of human PSCs-derived SRCs, both morphogen-directed differentiation and defined transcription factor (TF) over-expression were required to mediate the conversion [32]. Interestingly, engraftment was only achieved when HE precursor cells were programmed by TFs in vivo, suggesting that extracellular cues were essential for the subsequent specification of putative HSCs from hPSCs at this specific stage of cellular differentiation. Using iPSCs reprogrammed from AML patient skin fibroblasts [29] and BM cells devoid of somatic leukemic aberrations [33], we sought to generate putative SRCs by the development of HEs, and forced expression of previously defined TFs as a proof of concept for the use of PSCs for autologous HSCTs.

## 2. Material & Methods

### 2.1. Human iPSC Lines

The derivation of the human hiPSC-1 (normal iPSC from AML #15331 BM N1) and hiPSC-2 (normal iPSC from AML #28787 fib N18) used in this study has previously been described [29,33].

### 2.2. hPSC Culture

Experiments were performed using human ESC line (H9), hiPSC-1, and hiPSC-2, maintained on matrigel (BD, Mississauga, ON, Canada) in mouse embryonic fibroblast-conditioned media (MEF-CM) with 8 ng/mL basic fibroblast growth factor (bFGF), as previously described [34]. Media were changed daily and cells were passaged as clumps weekly using collagenase IV. In a subset of experiments, hPSCs were transitioned to mTeSR media (Stem Cell Technologies, Vancouver, BC, Canada) and maintained on matrigel with daily media changes. hPSC colonies cultured in mTeSR media were dissociate with 0.05% Trypsin for 5 min at 37 °C, pipetted thoroughly with p1000 to form small aggregates, and subsequently washed twice with PBS+2%FBS media for further experimentation (i.e., EB differentiation).

### 2.3. hPSC EB Differentiation

hPSCs were differentiated using two distinct methods: (1) using cytokines and BMP4 protocol in which hPSCs were treated with 200 U/mL collagenase IV (Invitrogen, Burlington, ON, Canada), scraped into clumps, and transferred into suspension culture to form embryoid bodies (EB), as previously described [34]; and (2) using cytokines and “supplemented StemPro-34” media in 10 cm plates of EZSPHERE (EZSPHERE TM, ASAHI GLASS CO; Well Size (µm) Diameter: 400–500, Depth: 100–200; No. of Well 14,000/dish) at a density of 5 million/dish, as previously described [32]. Experiments were conducted under normoxia (5% CO_2_ incubator) unless stated otherwise. In select cases, cells were maintained in hypoxic (5%O2/5% CO_2_/90% N2) culture conditions.

### 2.4. hPSC EHT Differentiation

Both MEF-CM-cultured hPSCs and mTeSR-cultured hPSCs were dissociated, as described above, on either EB day 10 or EB day 8, respectively (EHT day 0). Dissociated EBs were immediately processed for isolation of HE cells, as previously described [30]. Briefly, cells were resuspended in 1 mL of PBS+2%FBS and incubated with human CD34 MicroBead kit for 1 h (Miltenyl Biotec, Inc., Somerville, MA, USA; Cat# 130-046-702). After incubation, cells were washed again with PBS+2%FBS and human CD34^+^ cells were isolated by magnetic cell isolation (MACS) using LS columns (Miltenyl Biotec, Inc., Somerville, MA, USA; Cat# 130-042-401) according to the manufacturer’s instructions. Next, sorted human CD34^+^ cells were resuspend in supplemented StemPro-34 media, containing Y-27632 (10 μM), TPO (30 ng/mL), IL-3 (10 ng/mL), SCF (50 ng/mL), IL-6 (10 ng/mL), IL-11 (5 ng/mL), IGF-1 (25 ng/mL), VEGF (5 ng/mL), bFGF (5 ng/mL), BMP4 (10 ng/mL), and Flt-3L (10 ng/mL), hereafter called EHT media. All reagents were purchased from the suppliers listed in the referenced study. Cells were seeded at a density of 25–50 × 10^3^ cells per well onto thin-layer Matrigel-coated 24-well plates.

### 2.5. Lentiviral Gene Transfer

The following ORFs were purchased from Genecopoeia (GeneCopoeia, Inc., Rockville, MD, USA): (Table 1).

Upon personal communication and collaboration with Dr. Sugimura, RUNX1c (splicing variant 1, which is most recognized in hematopoietic development process) and ERG splicing variant 1 (NM_182918.3) were selected since some of the TFs previously published have multiple transcript variants (RUNX1, ERG, and LCOR) and were not specified in the publication. ORFs were not purchased for either RUNX1c or SPI1, but instead were obtained from Cord blood or reference cDNA. All TFs were first subcloned into a non-inducible pHIV backbone vector (Addgene; example pHIV(IRES)EGFP vector #21373, Addgene, Watertown, MA, USA) and then upon sequence verification further subcloned into the Lentiviral Tet-On 3G Inducible Expression Systems. Our TRE3G Vector system contains a TRE3G tetracycline-inducible promoter and our EF1a-Tet3G Vector expresses the Tet-On 3G transactivator protein from the human EF1 alpha promoter, enabling expression in the presence of tetracycline or the derivative of tetracycline, doxycycline (dox), when co-expressed. Lentivirus was produced from HEK 293FT cells with 2nd generation pMD2.G and psPAX2 packaging plasmids. Viral supernatants were harvested 72 h after transfection and concentrated by Amicon Ultra-15 (Ultracel-100) Centrifugal Filter Units (Millipore, Burlington, ON, Canada). The multiplicity of infection (MOI) was calculated by a dilution series on Hella cells. At day 3 of EHT culture, experimental cells were transduced with lentivirus in EHT culture media supplemented with Polybrene (8 μg/mL). All infections were carried out, as previously described [32], in a static volume of 250 μL in 24-well plate. The multiplicity of infection (MOI) for each transcription factor was 20. 12 h post-infection, and 250 μL of fresh EHT media was supplemented to dilute polybrene. Next, cells were either harvested for transplantation experiments (see xenotransplantation section) or parallel wells were kept for an additional three days of culture ± doxycycline (Sigma-Aldrich, St. Louis, MO, USA, Cat # D9891) to measure infection efficiency by percentage of positive fluorescent proteins by flow cytometry.

### 2.6. Xenotransplantation

Immunodeficient NOD.Cg-Prkdcscid Il2rgtm1Wjl/SzJ (NSG) mice were bred in a barrier facility and all experimental protocols were approved by the Animal Research Ethics Board of McMaster University. On the same day of HE cells transduction, mice were sublethally irradiated (315 rads) 12 h before transplantation. HE cells were transplanted by intra-femoral (IF) injection into each recipient NSG mouse, as previously described [24]. Experimental cell sources and cell doses for all transplants are discussed later. Mice were administered doxycycline in mouse drinking water (1 g/L doxycycline hyclate; Sigma, Cat # D9891) in light protected (tinted) bottles, which were replaced every few days, and via irradiated rodent diet food (0.625 g/kg doxycycline hyclate; ENVIGO, Indianapolis, IN, USA, Cat # 01306) for 2 weeks. We did observe that a combination of radiation and doxycycline (by food or water) lead to high morbidity rates and significant body weight loss (Figure S4B) and, therefore, we added 10 g/L sucrose to drinking water with doxycycline in combination with 0.5 mL saline subcutaneous injections for the first week post-transplant. To ensure mice maintained adequate doxycycline exposure and healthy body weights, mice were also daily gavaged with doxycycline. On day of harvest, BM from the injected (Inj) and contralateral (Ctl) femurs were collected separately and processed as previously described [25]. Cells were recovered separately by mechanical dissociation in IMDM Gibco (Thermo Fisher Scientific, Waltham, MA, USA) supplemented with 3% FBS (HyClone FBS, Mississauga, ON, Canada), and 1 mM EDTA (Invitrogen, Waltham, MA, USA). Immediately following harvest, RBCs were lysed using ammonium chloride, MNCs were counted, and then MNCs were analyzed by flow cytometry. BM cells from NSG mice injected IF with empty control vector (TRE3G-eGFP, Addgene, Watertown, MA, USA), were used as the negative controls. Both male and female mice were used as recipients and were distributed across all transplant groups, with no overt differences observed. No statistical method was used to predetermine sample size.

### 2.7. Colony Forming Unit (CFU) Assay

hPSC-derived HPCs were plated at 5.0 × 10^2^ cells/0.5 mL in Methocult H4434 (StemCell Technologies, Vancouver, BC, Canada) to assess clonogenic colony-forming unit (CFU) capacity, as previously describe [25]. Cells were incubated at 37 °C for 14 days and manually scored. Each CFU well represents an independent biological assay, as input cells and MethoCult formulations were individually prepared for testing in single wells. CFU were stained with calcein green (Invitrogen, Waltham, MA, USA) in Hank’s Buffered Salt Solution (HBSS) for 30 min and imaged with the Operetta High Content Imaging System (PerkinElmer, Guelph, ON, Canada).

### 2.8. Western Blotting

Cells were lysed with RIPA buffer (0.5% NP-40, 0.1% sodium deoxycholate, 150 mM NaCl, 50 mM Tris-Cl, pH 7.5) containing protease inhibitors. The protein concentration was determined using DC protein assay (Bio-Rad, Hercules, CA, USA), and equal amounts of protein were separated on 6–12% SDS-PAGE Gels and transferred to PVDF membrane (Invitrogen, Waltham, MA, USA). Nonspecific protein binding was blocked using either 3% Bovine Serum Albumin (BSA) or 5% powdered milk in Tris-buffered saline with 0.1% Tween-20 (TBST) at room temperature for 1 h followed by incubation with primary antibodies diluted in the blocking solution, overnight at 4 °C. The following morning, the membranes were washed with TBST and incubated with horseradish peroxidase-conjugated secondary antibodies (anti-mouse, NA931V, and anti-rabbit, NA934V, Amersham) for 1 h at room temperature. Membranes were then washed and imaged. Images were developed using the Immobilon Western Kit (Millipore, Burlington, MA, USA) and detected on a ChemiDoc imaging system (Bio-Rad, Hercules, CA, USA), and Bio-rad (version S.0.2.3.0, Rochester, NY, USA) was used to quantify protein content. GAPDH (1:10,000, no. ab9484, Abcam, Cambridge, United Kingdom) was used as a loading control. (Table 2).

### 2.9. RT-qPCR

Total RNA purification was performed using RNeasy Mini Kit (QIAGEN, Hilden, Germany), according to manufacturer’s instructions. Purified RNA was quantified on a Nanodrop 2000 Spectrophotometer (Thermo Scientific, Waltham, MA, USA). For RT-qPCR, cDNA was synthesized from 1 μg of total RNA using SuperScript III FirstStrand Synthesis System (Life Technologies, Carlsbad, CA, USA). RT-qPCR was carried out as previously described [35], using PowerUp SYBR Green Master Mix (Thermo Fisher Scientific, Waltham, MA, USA), utilizing manufacturer’s recommended cycling conditions on ViiA7 Real-time PCR system (Applied Biosystem, Waltham, MA, USA). (Table 3).

### 2.10. Conventional PCR

Genomic DNA was extracted by DNA Micro Kit (Qiagen, Hilden, Germany) following manufacturer’s protocol and analyzed by conventional PCR. (Table 4).

### 2.11. Flow Cytometry

For all live staining experiments, <1 × 10^6^ cells/200 μL were incubated with antibodies for 30 min at 4 °C, and then washed before flow cytometry. To exclude non-viable cells, 7AAD (Beckman Coulter, Pasadena, CA, USA) was used. Flow cytometry was performed using the LSRII Flow Cytometer with FACSDiva software (BD, Mississauga, ON, Canada) and analyzed by FlowJo software (version 10.8.0; BD, Mississauga, ON, Canada). (Table 5).

### 2.12. Gene Expression Profiling

Expression levels of RNA-seq and microarray data were obtained from series matrix sheets in the GEO repository (NCBI), described in the table below this section (Table 6). All data are available in a publicly accessible repository. Gene expression analysis was conducted using Partek Gene Suite (v6.6, Partek Inc., Chesterfield, MO, USA). Log2 transformation of RNA-seq data was completed, as previously described in *Nature* by Nakamura et al. [36], as a common technique to enable basic comparisons between RNA-seq and microarray datasets. Furthermore, the mean probe intensity was used for genes with multiple probes in the microarray data. Datasets were merged by common gene symbols. Batch effect was removed using Batch effect Remover in Partek Gene Suite across different studies that were selected as they also included FACS-purified cord blood phenotypic HSC samples tested in parallel with hPSC-derived cells. All genes were used in principal component analysis (PCA) and unsupervised hierarchical clustering. Gene set enrichment analysis (GSEA, Broad Institute, Inc., Massachusetts Institute of Technology, and Regents of the University of California, v4.0.3) was performed with default settings, using the gene expression matrix from Sugimura et al., from GEO (NCBI): GSE83719. Gene list names were obtained from the Molecular Signatures Database (MSigDB, Broad Institute, Inc., Massachusetts Institute of Technology, and Regents of the University of California) as grp files (Table 6).

### 2.13. Statistical Analysis

Data are presented as mean ± standard error of mean (SEM). Prism software (GraphPad Prism, San Diego, CA, USA, version 9.3.1) was used for all statistical analyses, and the criterion for statistical significance was *p* < 0.05. In all figures, n indicates biological replicates. Statistics are described in figure legends.

## 3. Results

### 3.1. Molecular Comparison of Human HE Derivation from Pluripotent Cell

**Figure 1:**
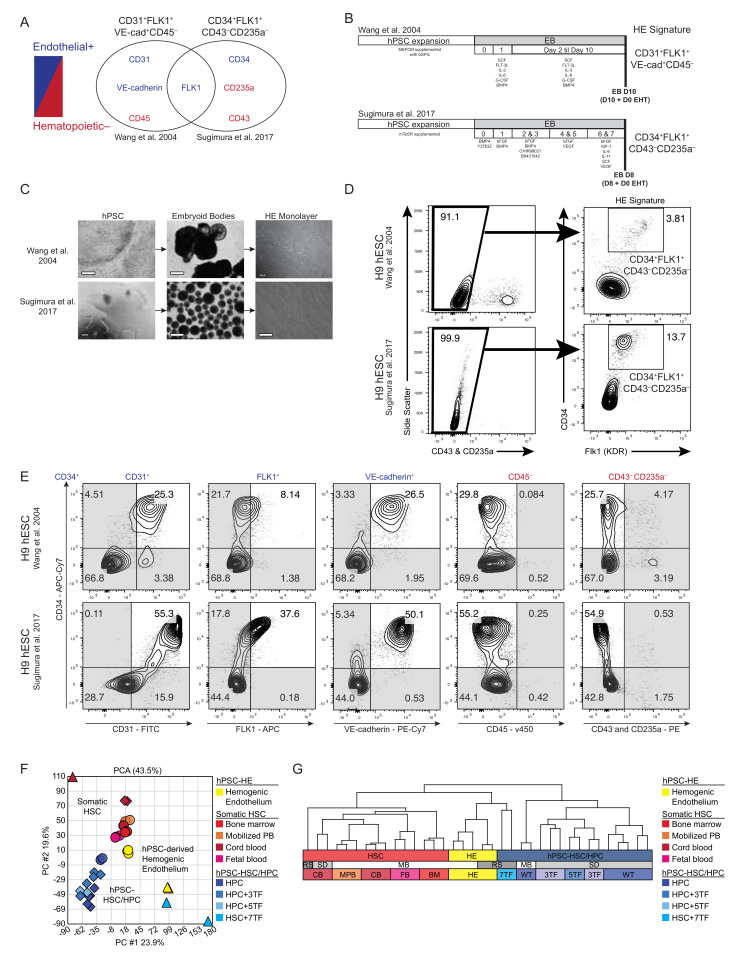
Co-expressed surface markers of hPSC-derived hemogenic endothelium. (**A**) Overlap of hemogenic endothelium (HE) phenotype with shared antigens from Wang et al. [38], and Sugimura et al. [32]. (**B**) Morphological comparison of HE derived from hPSC differentiated into embryoid bodies (EBs) using Wang et al. [38] (top), and Sugimura et al. [32] (bottom). Both protocols yield HE cells that adhere to the culture plate and grow post-dissociation of hEBs. White scale bar represents 500 mm; black scale bar represents 30 mm. (**C**) Timeline of HE derivation from hEBs using two distinct methodologies previously mentioned. (**D**) Flow cytometry of Sugimura et al., HE signature (CD34^+^Flk1^+^CD43^−^CD235A^−^) on differentiation day 10 generated using the Bhatia (Wang et al. [38]; top) and Daley (Sugimura et al., [32]; bottom) methods. (**E**) Overlap of HE phenotype with shared antigens combined in one flow cytometry panel on day 8 hEB (EHT day 0) derived from Sugimura et al. [32], protocol or day 10 hEBd (EHT day 0) Wang et al. [38], protocol. Despite distinct hEB culture conditions, both methodologies produce similar frequency of HE phenotypic markers. (**F**) Shared molecular signature of HE across Daley and Bhatia lab. A principal component analysis (PCA) correlation biplot comparing gene expression of FACS-purified HSCs (BM, N *n* = 3; CB, *n* = 7; FB, *n* = 3; MPB, *n* = 3; and hPSC(+7TF)-HSC, *n* = 2) and hPSC-HE(HE, *n* = 2) and hPSC-HPCs (wt, *n* = 7; 3TF, *n* = 3; and 5TF, *n* = 2) from a combined dataset including GSE83719 (Sugimura et al. [32]) and GSE49938 (Doulatov et al. [30]). (**G**) Unsupervised hierarchical clustering of samples described in F. Abbreviations (top row): hematopoietic stem cell, HSC; hemogenic endothelium, HE; in vitro human pluripotent stem cell-derived hematopoietic progenitor cells, in vivo human pluripotent stem cell-derived hematopoietic stem cell, hPSC-HSC/HPC; (middle row): Mick Bhatia, MB; Ryohichi Sugimura, RS; Sergei Doulatov, SD; (bottom row): cord blood, CB; mobilized peripheral blood, MPB; fetal blood, FB; bone marrow, BM; hPSC-HE, HE; hPSC(+7TF)-HPC, 7TF; wildtype, WT; hPSC(+3TF)-HPC, 3TF; hPSC(+5TF)-HPC, 5TF.

Since no distinguishing cell surface markers have been described to identify HE [37], yet transcription-factor-mediated expression in hPSC-derived HE has recently been shown to confer the ability to generated SRCs [32], we sought to directly compare the phenotypic markers of previously characterized primitive endothelial-like cells. Specifically, we compared cells that are responsible for hematopoietic output, and thus functionally represents hemogenic precursors arising from hPSCs [38] to a more recent derivation approach utilized by Sugimura et al. (Figure 1A) [32], relying on EB formation [39,40]. HEs have previously been shown to be detectable as early as day seven of EB development, and have been defined as PECAM-1-, FLK-1-, and VE-cadherin-positive endothelial cells that also lack CD45 expression (PFV^CD45−^) HE cells [38,41,42]. In comparison, utilizing an adapted protocol to derive HE from hPSCs that was previously verified to have hematopoietic potential [39], HE cells are phenotypically characterized by the FLK^+^CD34^+^CD43^−^CD235A^−^ expression by Sugimura et al. (Figure 1B) [32]. PFV^CD45−^ cells had one overlapping expression marker with HE derived by Sugimura et al., FLK-1 (fetal liver kinase 1, also known as a kinase insert domain-containing receptor, KDR and vascular endothelial growth factor receptor 2 (VEGFR2) (Figure 1A) which has previously been described as a positive marker for HE cells [43]. Notably, PFV^CD45−^ cells also have more phenotypes associated with endothelial surface markers (Figure 1A) in comparison to HE cells derived using an alternative PSC differentiation methodology (Figure 1B). Side-by-side morphological comparisons of hPSC-derived hemato-endothelial progenitor cells revealed a similar monolayer after EB dissociation and culture (Figure 1C). Methods to derive PFV^CD45−^ cells capture the HE signatures of FLK^+^CD34^+^CD43^−^CD235A^−^ cells (Figure 1D) [38], though to a lesser extent. However, the means to select HEs using the PFV^CD45−^ provides a potentially superior approach to purification amongst differentiating hPSCs using positive selection vs. less definite negative selection based on the absence of the marker. To this point, phenotypic expression was analyzed and all surface markers by flow cytometry were common of HEs, independent of the methodology used for derivation (Figure 1E); however, higher CD31/VE-cadherin markers were observed with HE derived using Sugimura et al.’s methodology across experimentations (Figure S1A). These observations remained the same under hypoxic conditions of HE derivation (Figure S1B), which has previously been suggested as an important state in adult BM HSC niches to defend against oxidative stress [44,45,46], but in fact when quantitively analyzed, has no bearing on the molecular phenotype of HEs differentiated from hPSCs. To determine whether global transcriptome analysis would demarcate these HE populations, we next used gene expression profiles from hPSC progenies from several sources and procedures, including 7TF overexpression [30,32,47], and compared these to publicly available datasets of primitive hematopoietic populations enriched for HSC/HPCs (CD45^+^CD34^+^CD38^−^), including BM, MPB, CB, and fetal blood (FB) sources (Figure 1F). Despite being derived using different in vitro differentiation protocols, PFV^CD45−^ HE cells clustered with HE cells previously reported from other groups (Figure 1G), suggesting that human hPSC-derived HEs are functionally and molecularly fairly similar, independent of differentiation methods which merely change the efficiency and frequency of the derivation from hPSCs (Figure 1E). Furthermore, 7TF hPSC-HSPCs clustered closer to HE cells than to hematopoietic stem and progenitor cells (HSPCs), implying an incomplete conversion to an HSC-like state (Figure 1G). As a positive control, using the gene expression matrix provided by Sugimura et al., GSEA analysis comparing the genes of interest in the key cell types of interest were reproduced. Similar to their previous findings, Figure S1B shows that the 7TF hPSC-HSPCs are more highly enriched than hPSC-derived HEs for Integrin genes. Additionally, using two well-established gene lists for HSCs [48,49], we found that both gene lists were highly similar in their results and were more highly enriched in CB than 7TF and HE (Figure S1B). Interestingly, the difference between CB and HE was smaller than the difference between CB and 7TF. Furthermore, HE cells were more highly enriched in HSC genes than the 7TF hPSC-HSPCs, again suggesting an incomplete conversion of 7TF to an HSC-like state (Figure 1G and Figure S1B). Based on these results, we sought to apply these methods of HE derivation from hPSCs toward a proof-of-principle experimentation to determine if normal HSPCs can be generated from hiPSCs obtained from AML patient cells devoid of leukemic aberrations [29] upon 7TF overexpression [32].

### 3.2. Derivation of HE from AML Patient-Specific iPSCs into Endothelial-to-Hematopoietic Transition (EHT) Conditions for Forced 7TF Expression

**Figure 2:**
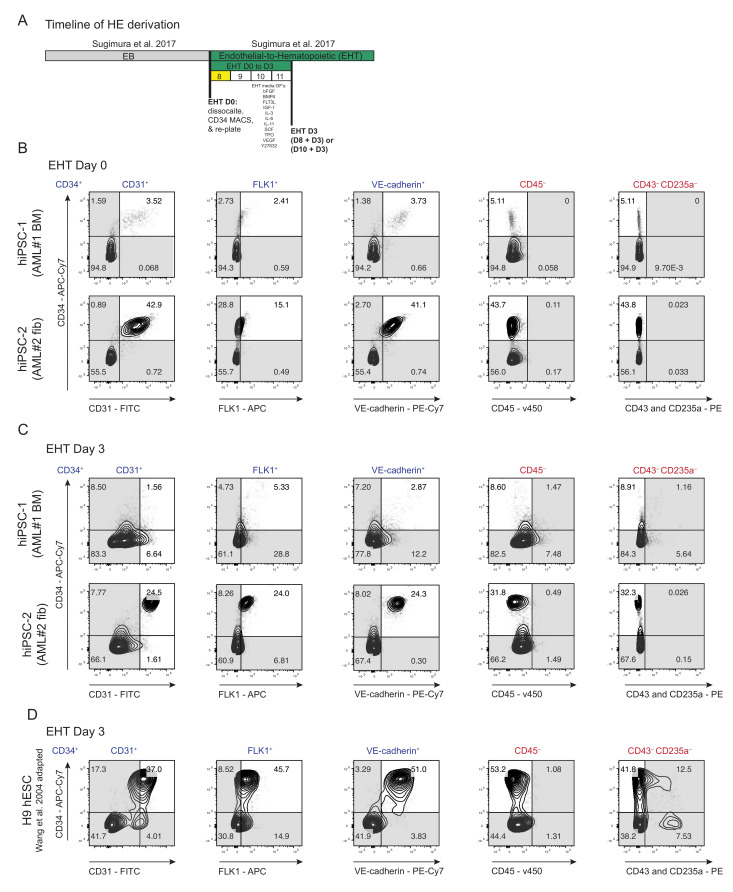
Endothelial-to-hematopoietic transition (EHT) of AML patient-specific iPSCs. (**A**) Schematic depicting simplified timeline of HE derivation used on various hPSCs. HE cells were isolated through dissociation of hEBs and enriched by positive selection of CD34^+^ through Magnetic-Activated Cell Sorting (MACS) at day 8 (or day 10) and then further cultured in Endothelial-to-Hematopoietic Transition (EHT) medium for the indicated number of days (Sugimura et al. [32]). (**B**) Flow analysis of HE phenotype on EHT day 0 post-CD34^+^ MACS enrichment in two AML-iPSC lines, hiPSC-1 (AML-iPSC derived from reprogramming AML patient 15331 bone marrow cells) and hiPSC-2 (AML-iPSC derived from reprogramming AML patient #2 fibroblast cells). (**C**) Flow analysis of HE phenotype on day 3 of EHT on hiPSC-1 and hiPSC-2. (**D**) HE phenotype of hESC line H9s derived using Wang et al. [38], methodology depicted on one flow cytometry panel.

In cases where AML patients fail chemo-induced remission, HSC transplantation provides an impactful treatment option [50,51,52]. As such, the generation of AML patient-specific HSCs that are devoid of the leukemic aberration(s) would provide an ideal source in which to obtain a healthy autologous blood source for transplantation. The potential of using reprogramming to generate healthy HSCs from an AML patient has yet to be explored and it remains unclear whether the generation of AML-patient HSCs is even possible or capable of bestowing surrogate properties such as SRC capacity. By definition, HE is a transient, specialized endothelium with the capacity to generate hematopoietic cells through a gradual process of endothelial-to-hematopoietic transition (EHT) [53,54]. Based on this, we evaluated the potential of generating normal HSPCs from AML patient-derived iPSCs adhering to the specific methods used by Sugimura et al., in which HE cells are cultured in EHT medium and have previously been shown to be conducive to TF reprogramming (Figure 2A) that resulted in SRC activity [32]. We selected two patient samples (AML 15331 and 28787) to generate iPSCs [29,33] for use in this study. AML genetic interrogation of ≥4 iPSC lines per sample previously revealed that all colonies were derived from blood cells (AML 15331) or skin fibroblasts (AML 28787) devoid of leukemic aberration t(9;11)(p22;q23) [55], and thus were classified as normal iPSCs derived from AML patients. Therefore, they represent an ideal source in which to interrogate the potential of deriving autologous HSCs and progenitors. We found that both iPSC lines produced HE phenotypes (Figure 2B), though hiPSC-2 yielded higher frequencies of positive HE phenotypes CD31, FLK1, and VE-cadherin in comparison to hiPSC-1 at EHT day 0 (Figure S2A). To accurately control the quality of HE cells derived using our normal iPSCs derived from AML patients upon EHT induction, we reassessed both the HE panels of phenotypes (a combination of FLK^+^CD34^+^CD43^−^CD235A^−^ cells and our PFV^CD45−^ cells). Consistent with Sugimura et al., we routinely observed an HE phenotype in both iPSC lines upon CD34^+^ population enrichment based on magnetic cell isolation and an extended culture with hematopoietic cytokines that are believed to encourage EHT on fibronectin-coated plates (Figure 2C; EHT day 3). These results provided a platform in which we were confident in interrogating whether normal iPSCs derived from AML patients can produce SRCs devoid of leukemia-associated aberrations upon the forced expression of specific TFs having successfully derived an HE phenotype.

PFV^CD45−^ HE cells can be cultured and expanded for a length of time, but unfortunately, produce hematopoietic progenitors incapable of engraftment potential [24,38]. Thus, having demonstrated that hESC-derived HEs are functionally and molecularly similar, independent of differentiation methods prior to EHT induction (Figure 1), we adapted our protocol and designed a merged approach whereby we performed our classical method of hematopoietic differentiation [38] followed by subsequently culturing in EHT medium, as described previously [32], using hESCs (Figure S2B). We questioned whether adapting our methods would bestow long-term engraftment upon TF reprogramming on hESC-derived HE cells, having previously yielded negative potential [24,38]. Cells were culture and EB formed as previously described [34]. However, we isolated HE based on the magnetic cell isolation of a CD34+ population at day 10 of EB formation (EHT day 0; Figure S2C), and then subsequently cultured in EHT medium for 3 days. Using this adapted protocol, we found similar levels of enrichment for the HE phenotypic panel on EHT day 3 (Figure 2D). Moreover, the phenotypic expression remained unaltered under hypoxic conditions (Figure S2D) consistent with our previous results (Figure S1B). Overall, our results show that both methodologies, either mirroring the protocol directly from Sugimura et al., (Figure 2A) or our newly adapted protocol (Figure S2B), derive similar phenotypic HE cells (Figure 2C,D) from hPSCs, suggesting that both outcomes of in vitro EHT are conducive to TF reprogramming.

### 3.3. Generation and Forced Expression of 7TF under HE In Vitro Differentiation Conditions from hPSCs

**Figure 3:**
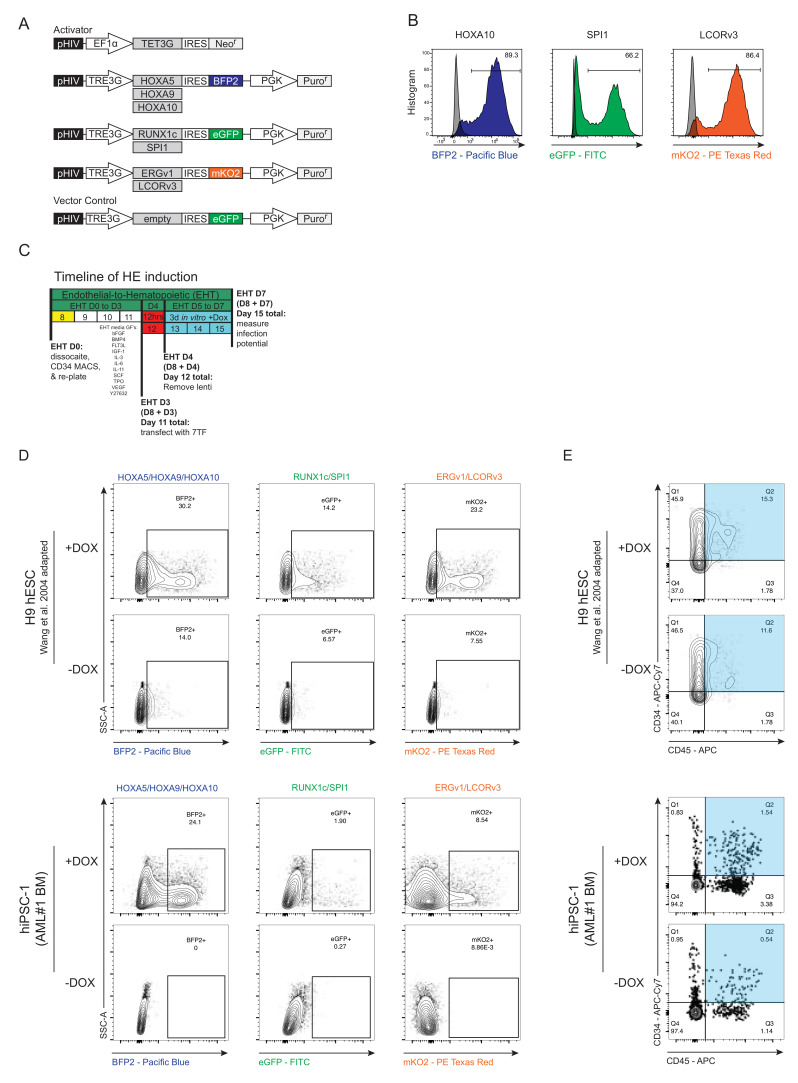
Generation and forced expression of 7TF under HE conditions in AML-iPSCs. (**A**) Schematic representation of the Doxycycline-inducible system utilized throughout experiments with a defined set of seven hematopoietic transcription factors (TF). Human TFs were cloned into pHIV-TREG vector, which has an internal ribosome entry site (IRES) translation link to distinct fluorescent proteins BFP2, mKusabiraOrange2 (mKO2), or eGFP. (**B**) Doxycycline induction of individual TFs in HE cells. Fluorescent proteins are representative single stains of select TFs. (**C**) Schematic depicting temporal forced expression of 7TF on HE derived from hPSC in the presence of doxycycline in EHT medium maintained in vitro, as well as empty vector control. (**D**) Representative transduction efficiency of TFs comprising all three fluorescent protein channels (Wang et al. [38]). (**E**) Doxycycline induction of 7 TF-transduced HE, maintained in vitro 3 days beyond the day of transduction. Acquisition of a larger hematopoietic phenotype (CD34^+^CD45^+^) observed in HE cells treated with Doxycycline vs. untreated cells (Wang et al. [38]).

Based on the ability to generate HE from both normal iPSCs from AML patients and hESCs, along with the recent breakthrough study reported in *Nature* [32] which recapitulated the EHT lineage transition from HE to generate SRCs, we hypothesized that the introduction of these defined HSC-specific TFs may endow hPSC-derived HE with the potential to engraft multi-lineage hematopoiesis in vivo. Thus, we subcloned the following TFs: *RUNX1c*, *SPI1*, *HOXA5*, *HOXA9*, *HOXA10*, *ERG*-transcript variant 1 (*ERGv1*; shorter), and *LCOR*-transcript variant 3 (*LCORv3*; longest) into a lentiviral Tet-On 3G inducible expression system. These resulting vectors were validated by biological testing for expression and transduction into target cells (Figure 3A and Figure S3). These TF constructs were carefully engineered with distinguishing fluorescent proteins (FPs: eGFP; BFP2; and mKusabiraOrange2, mKO2) to be co-expressed with individual TF constructs due to the presence of an internal ribosome entry site (IRES) cassette, making our system a unique inducible reporter system by the inclusion of doxycycline (Dox). Lentiviral ectopic expression of select individual TFs demonstrated functional expression validity (Figure 3B). In order to include hematopoietic phenotyping, in addition to multiplexing our fluorescent reporter system, we categorized the 7TFs into groups utilizing three FPs: (1) the likely essential two TFs *RUNX1* and *SPI1* (green, eGFP), based on overlapping TFs used between the Sugimura et al. and Lis et al. groups; (2) the HoxA genes, *HOXA5*, *9*, and *10* (blue, BFP2); (3) and *ERGv1* and *LCOR* (orange, mKO2), both associated with oncogenesis [56,57,58,59]. We transduced hPSC-derived HE cells at day 3 of EHT culture for 24 h, and subsequently added fresh EHT medium supplemented with dox (Figure 3C). The induction of TF expression post-transduction was successful (Figure 3D), and the concomitant emergence of CD34^+^CD45^+^ hematopoietic cells occurred (Figure 3E). These results demonstrate that transduction with a defined set of 7TFs promotes a hematopoietic phenotype from hPSC-derived hemogenic endothelium, independent of the methodologies used. Additionally, these results confirm the validity of the systems and provide the foundation for interrogating the biology and mechanism of TF regulation for hPSC-guided development towards hematopoietic progenitor capacity.

### 3.4. Progenitor Capacity from hPSC-Derived HE with 7TF Expression

**Figure 4:**
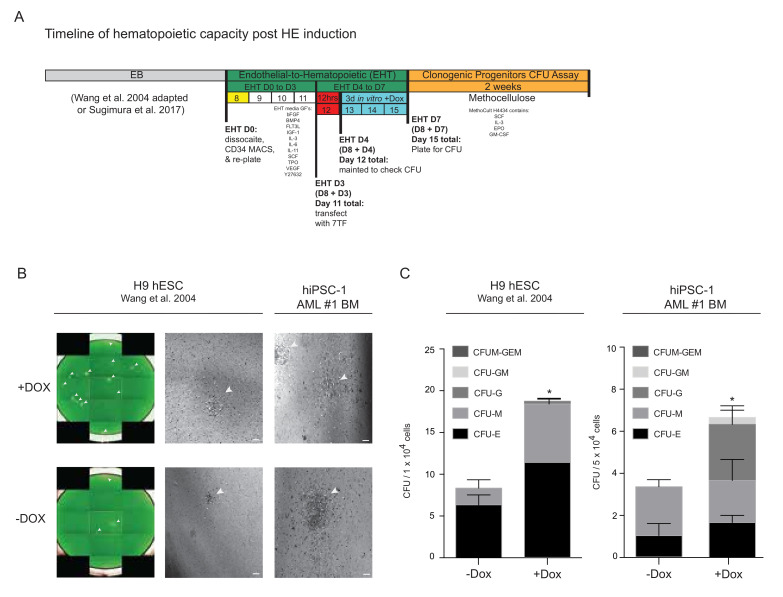
Progenitor capacity from hPSC-derived HE upon 7TF initiation. (**A**) Schematic depicting temporal forced expression of 7TF to assess clonogenic progenitor or “colony forming unit” (CFU) assay on HE derived from hPSCs (Sugimura et al. [32] and Wang et al. [38]). (**B**) Representative whole-well CFU images of hPSC lines stained by calcein-green (475 ex; 525 em) fluorescence on day 14 of cultures. Images were acquired at 2× using Operetta High Content Screening (Perkin Elmer) by means of calcein-green am staining. Whole-well images were stitched in Columbus Image Data Storage and Analysis System version 2.9.0 (Perkin Elmer). Scale bar 2 μm. White arrow heads highlight colonies formed (Wang et al. [38]). (**C**) Total number of hematopoietic colony forming units (CFUs) and number of colony subtypes CFU-Erythroid, CFU-Granulocyte, CFU-Monocyte/macrophage, and CFU-GM and CFU-GEMM (Wang et al. [38]). Unpaired Student *t*-test was performed for statistical analysis * = *p* < 0.05. All data shown are mean ± SEM (N = 3–4) .

To investigate the functional outcome of the temporal expression of the 7TFs under EHT conditions, we transduced HE cells, as detailed in Figure 3, and assayed for the clonogenic progenitor capacity after 3 days of culture post-exposure to the delivery virus. As previously described, we compared two distinct protocols and approaches: (1) Sugimura et al. and (2) our adapted protocol which encompassed merging methods (Wang et al. [38] adapted) to derive hemogenic endothelium from hPSCs on normal iPSCs derived from AML patients and hESCs, respectively (illustrated in Figure 4A). As predicted, we routinely observed enhanced hPSC-derived hematopoietic colony-forming unit (CFU) morphology in hPSC HE-derived cells treated in the presence of dox (Figure 4B) when cultured for 14 days in vitro in both hESC and AML-iPSC. To control for variations in the CD34^+^CD45^+^ frequency (Figure 3D), we determined the total CFU output and lineage distribution per 40,000 cells seeded. A higher functional progenitor capacity was observed in both hPSC lines (H9s and BM-derived normal AML-iPSC) transduced with the 7TF in the presence of dox (Figure 4C). These results indicate that AML iPSCs possess a normal differentiation capacity towards the hematopoietic progenitors and lineage development, and 7TF expression augments the putative hematopoietic progenitor output from iPSCs derived from AML-patient fibroblasts. Based on this successful induction to increase the progenitor output from AML-derived iPSCs, we sought to measure the in vivo SRC activity using these established methods and approaches.

### 3.5. SRC Engraftment Potential of TF-Induced Hemogenic Precursors Derived from Healthy iPSCs Established from AML Patients

**Figure 5:**
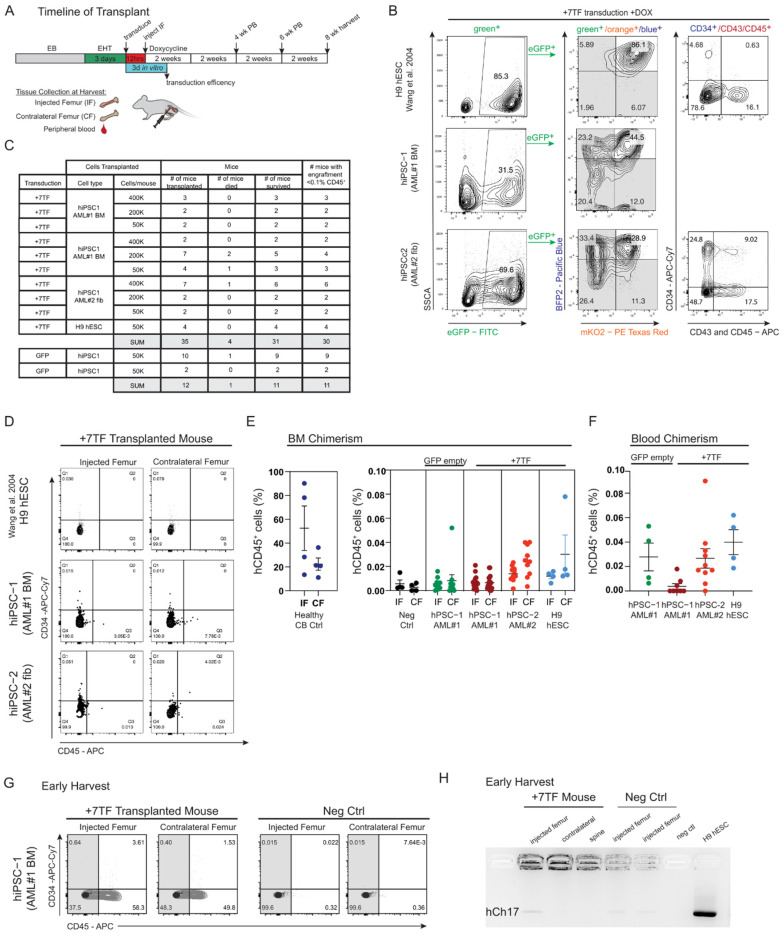
Early harvest of transplanted mouse reveals human hematopoietic chimerism. (**A**) Schematic depicting timeline of transplantation experiments. Hemogenic endothelium cells infected at day 3 EHT were incubated for 24 h and intrafemorally (IF) injected into mice. Doxycycline was provided for 2 weeks in vivo after transplantation into sub-lethally irradiated immune-deficient NSG mice. Three days post-transfection, transduction efficiency of transplanted cells were assessed. Tissues collected during each harvest was as follows: injected femur, contralateral femur, and peripheral blood via cheek bleeds. (**B**) Representative transduction efficiency flow plots of fluorescent proteins multiplexed by flow cytometry. Doxycycline induction of 7 TF-transduced hemogenic endothelium, maintained in vitro 6 days beyond the day of transplant. Acquisition of hematopoietic phenotype (CD34^+^CD45^+^) observed post-transduction (Wang et al. [38]). (**C**) Table summarizing the numbers of mice, cell doses transplanted, and chimerism outcome. (**D**) Representative flow plots of bone marrow of NSG mouse engrafted with HE-7 transcription factor analyzed at 8 weeks for scid-repopulating cells (hCD34^+^hCD45^+^). N numbers represent transplanted mice (Wang et al. [38]). (**E**) BM chimerism of NSG mice engrafted with HE-7 transcription factor analyzed at 8 weeks for human hCD45+. Data shown as mean ± SEM (N = 3–18); each dot represents a separate mouse. Mice transplanted with cord blood (CB) were harvested at 6 weeks during a separate round of experimentation. Mice that were not transplanted with hPSCs are referred to as negative control (neg ctrl). Mice that were transplanted with HE transduced with an empty eGFP vector are referred to as hPSC-1 + eGFP. (**F**) Blood chimerism of NSG mice engrafted with HE-7 transcription factor were analyzed at 8 weeks by cheek bleeds. (**G**) Representative flow plots showing human chimerism after transplant. Human hematopoietic phenotyping of 7TF-transplanted mouse harvested at day 12, in parallel with a negative control mouse. (**H**) Genomic DNA extracted from harvested tissue and probed for human sequences by conventional PCR (hCh17; alpha-satellite chromosome 17).

While we have previously verified that AML patient-derived iPSCs can generate hematopoietic progenitors with an in vitro myeloid lineage maturation capacity [29,33], multi-lineage reconstitution potential could not be evaluated in vivo, due to a lack of bona fide HSC generation methods available at the time. Similarly, we have historically been unsuccessful in deriving the long-term engraftment of SRCs using hESCs [25]. Using our inducible transgene system (Figure 3), hPSC-derived HE cells were infected on day 3 of EHT culture with 7TFs, and after 24 h, the transduced cells were injected intrafemorally into sub-lethally irradiated immune-deficient NOD LtSz-scidIL2Rγnull (NSG) mice to interrogate hPSC-derived SRCs’ potential. As illustrated in Figure 5A, mice received doxycycline in their drinking water and diet for 2 weeks to induce transgene expression, after which doxycycline was withdrawn and hematopoietic chimerism was assessed over time. All mice were subsequently harvested at 8 weeks post-transplantation and the injected femur (IF), contralateral femur (CF) BM, and peripheral blood (PB) were assessed to determine any potential migration capacity in vivo (Figure 5A).

To assess the induction efficiency of the 7TF in cells that were transplanted into recipient mice, a portion of the cells remained in the culture post-lentiviral removal and were phenotypically characterized by flow cytometry. FP expression, indicative of TF expression and hematopoietic frequency, were measured at EHT day 7, and demonstrated an expression of TFs as well as an emergence of a CD34^+^CD45^+^ phenotype (Figure 5B). Next, we transplanted three sets of HE-7TF-derived cells obtained from either normal iPSCs derived from AML patients using the Sugimura et al., protocol (hiPSC-1 and hiPSC-2), or HE-7TF-derived cells obtained from the hESC using our adapted protocol (H9 cell line; Figure S4A). Additionally, a control set of HE cells were transplanted in which HE cells were transduced with an empty control vector. It was previously reported that the stem cell frequency of HE-7TF cells was approximately 1 in 10,000 cells, calculated by a limiting dilution assay using the software ELDA [32]. Thus, we transplanted varying levels of HE-7TF-derived cells, ranging from 50,000 cells up to 400,000 cells, encompassing a total of 35 experimental mice and 12 control mice (Figure 5C). Early in the experimentation, we observed some mice with a low body weight and determined that radiated mice were not drinking sufficient water, potentially as a side effect of radiation sickness in conjunction with supplementing dox in their diet (Figure S3B) [60], occasionally resulting in premature death (Figure 5C). We thus decided to supplement mice with 1mg/mL doxycycline hyclate and 10 mg/mL sucrose via daily gavage to ensure mice obtained a sufficient dose of dox during the two-week administration, as well as avoiding endpoint monitoring based off a significant drop in the mouse’s body weight, relatively to its starting weight (see methods section for additional information). Human engraftment was evaluated after 8 weeks using human CD34 and CD45 antibodies from the injected femurs and contralateral femurs of recipient mice. The frequency of human hematopoietic cell chimerism was extremely rare and could not be fully captured by flow cytometric analysis across all hPSC HE-7TF-derived cells (Figure 5D). Similarly, when blood chimerism was assessed at both four weeks and six weeks, no human hematopoietic cell chimerism was observed (data not shown). One mouse did display human CD45^+^ detection (Figure S4C), though the levels and frequency were not on par with previous reports [32]. Notably, CB cells transplanted in a separate experiment in the same IF manner displayed significant engraftment potential in comparison to all sets of HE-7TFs when evaluating total human CD45^+^ BM chimerism (Figure 5E; separate scale used to highlight striking difference in chimerism). All sets of HE-7TFs yielded engraftment levels indistinguishable from the negative control mice, in which no cells were transplanted, but BM was harvested as an additive control measure (Figure 5E; black vs. all other colors). Similarly, blood chimerism produced next to no human CD45^+^ detection (Figure 5F), suggesting little to no migration potential capacity in vivo. In lieu of the previously described radiation sickness, one mouse had to be harvested early to due significant weight loss and resulted in a singly intriguing yet unreproduced result. Human hematopoietic phenotyping of one 7TF-transplanted mouse, harvested at the ethical endpoint on day 12 post-transplant, demonstrated striking human CD45^+^ BM chimerism in both its injected femur and contralateral BM (Figure 5G). Impressively, HE-7TF cells derived from hiPSC-2 displayed a human origin when assessed by PCR (Figure 5H), in which a faint band of human chromosome 17 was detected, in comparison to DNA extracted from the BM of a negative control mouse that was harvested in parallel. Unfortunately, HE-7TF cells derived from hiPSC-2 were not sufficient to confer a similar level of detection in the contralateral femur, or in any other mouse, or at any longer timepoints. Notably, it was not possible to confirm that we completely reproduced all aspects of the original report due to variation in both the targeted cell type, e.g., AML patient-derived iPSCs, and variation in the transduction efficiencies and stoichiometry of TF expression that was not fully described previously. Overall, our results suggest a deficient acquisition of functional SRCs from hPSC-derived hemogenic endothelia upon transcription factor induction independent of the methodology used to derive the cells using various sources of hPSCs.

## 4. Discussion

Since their discovery, human embryonic stem cells (hESCs) have offered great promise as a near-unlimited source of a variety of therapeutically relevant cell types, due to their ability for indefinite self-renewal and their potential to form all somatic cell types [61]. Subsequently, the derivation of hiPSCs from adult somatic cells not only established a paradigm shift in our understanding of the developmental potential of terminally differentiated cells in demonstrating the ability to drastically be altered by a relatively simple genetic approach, but has also provided a foundation for novel autologous cell-based therapies to be explored [20,62]. Over the years, extensive investigations of new methods for the derivation of HSCs from hPSC sources have suggested that robust transplantable HSCs were around the corner. The first report on such attempts showed limited success of HSC properties from resulting primitive hematopoietic cells [24]. Almost 20 years later, various groups have reported dozens of methods and approaches for the derivation of HSCs from hPSCs, including complex developmental programs of definitive and primitive hematopoiesis, co-culture methods, embryonic hematopoietic phenotypes, and sequential growth factor treatments to mimic native niches and unique regulating signaling cascades [21,22,23,25,63,64,65,66,67,68]. Despite the augmentation of hematopoietic progenitors, accelerating timing, and ease of protocols, e.g., less expensive and numerous factors, those studies capable of testing the SRC/HSC capacity consistently remained unsuccessful. This left the clinical prospects of therapeutic benefit elusive. Our current study further demonstrates that the current understanding of HSC genesis from hPSCs remains naive and is incapable of discerning methods for the pre-clinical and clinical study design for HSCs from hPSCs. In addition, we propose the field question the current understanding of the causal and functional impact of published regulators of hematopoietic specification from hPSCs that is not restricted to developmental biology. This is supported by our experience in human PSCs that is similar to murine PSCs where bone-marrow-derived HSCs are still used for the study of HSC biology due to the inconsistency and difficulty of deriving HSC from PSCs, even in the mouse system.

Despite the failures in demonstrating robust HSCs from hPSCs, procedures aimed at mimicking in vivo niches have had some limited success. Serially transplantable hPSC-derivatives have been reported, although with modest levels of chimerism and a lack of definitive hematopoiesis, as judged by primitive erythroid characteristics and limited lymphoid potential [23,66,69]. Two independent groups have reported a derivation of putative HSC-like cells from hiPSCs utilizing iPSC-derived teratomas as in vivo bioreactors [70,71]. Both studies combined cytokine treatment and OP9 stromal cell co-injection to recapitulate the BM niche environment more closely. Teratoma-mediated hematopoiesis generated derivatives bearing the hallmarks of bona fide HSCs, namely sustained engraftment, and the lympho-myeloid differentiation potential after BM homing in xenograft assays. These combined studies were not successful at demonstrating the two hallmark properties of HSPCs, but both strongly contributed to the overall goal of generating hPSC-HSPCs by suggesting a crucial role of the niche in providing inductive cues for functional HSPC development.

Most recently, the transcription factor-mediated specification of HSPCs has been extensively studied. Reports utilizing murine fibroblasts identified a combination of four transcription factors (Gata2, Gfi1b, cFos, and Etv6) capable of inducing endothelial precursors that, upon culture modifications, generated HSPC-like cells, although the engraftment potential of the resulting cells was not evaluated [72]. Additional studies have focused on either lineage-restricted precursors or committed progenitors of the hematopoietic system as the starting cell population for reprogramming. A hybrid study, combining hPSC differentiation into precursor cells with a subsequent re-specification of the resulting cells, described a set of five transcription factors (HOXA9, ERG, RORA, SOX4, and MYB), which imparted self-renewal and multilineage potential in vitro and short-term engraftment potential in vivo on these cells [30]. Murine committed lymphoid/myeloid progenitors and myeloid effector cells have been successfully reprogrammed into induced-HSCs (iHSCs) utilizing the transient expression of eight transcription factors (Run1t1, Hlf, Lmo2, Prdm5, Pbx1, Zfp37, Mycn, and Meis1) [73]. The resulting cells exhibited self-renewal and multilineage differentiation potential at the clonal level and were serially transplantable [73]. Another study exploited the current underlying ideas behind the endothelial-to-hematopoietic transition that results in the formation of definitive HSCs within the developing embryo to generate human multipotent progenitors (hMPPs) from non-hemogenic endothelial cells using four transcription factors (FOSB, GFI1, RUNX1, and SPI1) [74]. Reprogramming was critically dependent on the instructions provided by the specialized serum-free vascular niche; generated hMPPs were capable of long-term primary and secondary multilineage engraftment [74]. Notably, two breakthrough studies reported in *Nature* recapitulated the EHT lineage transition from HE to generate putative functional mouse and human HSCs, from the Rafii and Daley groups, respectively, that were able to reconstitute recipient mice [31,32]. In the case of human PSC-derived HSPCs, both morphogen-directed differentiation and defined TF over-expression were required to mediate the conversion [32]. Interestingly, engraftment was only achieved when hPSC-HE precursors were programmed by TFs in vivo, again suggesting and fully consistent with unidentified extracellular cues that are essential for the subsequent specification of hPSC-to-HSPCs. When taken into consideration that independent groups have reported a limited derivation of hPSC-HSPCs in vivo using teratoma formation, and now, most recently, HSC engraftment of 7TF-HE cells being achieved by in vivo programming, this further highlights that extracellular cues from the in vivo BM niche are crucial.

Overall, our study reveals the potential of hPSCs to generate bona fide HSPCs, but highlights our lack of understanding of how these TFs act, on which cell types they act on (e.g., HSC vs. common lymphoid progenitors or common myeloid progenitors), and the nature of niche signals that direct the specification of HSPCs from hPSC-derived HE cells. Importantly, as not all aspects can be reproduced from one report to another report identically, we would like to emphasize that our observed results included here do not dismiss the studies of Sugimura et al., but rather our own work found it difficult to generate SRC using a similar model system and approach. Moreover, although our results show that both methodologies derive phenotypic HE cells (Figure 2) from hPSCs, and both outcomes of in vitro EHT were conducive to TF reprogramming, we recognize that in vivo experimentation would have potentially been informative using H9 hESCs, used by Sugimura et al., as we initially did in the experimental results shown in Figure 1. Since H9s were not used in the same manner as for Sugimura et al., for various in vitro and in vivo experimentations, as this was not the goal or target of the authors in our current study, we cannot conclude that our in vitro culture or lenti-viral system contributed to negligible in vivo chimerism using iPSCs derived from AML patients.

## 5. Conclusions

We propose that more emphasis should be placed on methodological improvements, including robust data collection and evaluation, together with the complete disclosure of protocols and publication of all outcomes, be that favorable or negative results. Experimental replication (as best as possible) and validation should be achieved at the pre-clinical stage to maximize the prospects of successful clinical translation.

## Figures and Tables

**Table 1:** Transcription Factor ORFs.

Description	Biological (Y/N)/BSL Level	Cat #
ERG (transcript variant 1) ORF clone	Y, level 1	EX-Z1500-Lv165
LCOR (transcript variant 3) ORF clone	Y, level 1	EX-E2088-Lv165
HOXA10 ORF clone	Y, level 1	EX-Z5789-Lv165
HOXA5 ORF clone	Y, level 1	EX-F0180-Lv165
HOXA9 ORF clone	Y, level 1	EX-P0078-Lv165

**Table 2:** Primary antibodies.

Antibody	Company	Cat #
Rb mAb to RUNX1	Abcam	ab92336
Rb mAb to PU.1/SPI1	Abcam	ab76543
Rb mAb to HOXA5	Abcam	ab140636
HOXA9 (rabbit polyclonal IgG)	EMD Millipore Corp	07–178
HOXA10	Abcam	ab191470
Rb mAb to ERG	Abcam	ab92513
Rb mAb to LCOR	Abcam	ab171086
Ms mAB to GAPDH	Abcam	ab8245

**Table 3:** Primers.

Gene Name	Gene Forward (5′–3′)	Reverse (5′–3′)
RUNX1c	CGT ACC CAC AGT GCT TCA TGA	GGC ATC GTG GAC GTC TCT AGA
SPI1	GCC AAA CGC ACG AGT ATT ACC	GGG TGG AAG TCC CAG TAA TGG
HOXA5	ACC CCA GAT CTA CCC CTG GAT	CGG GCC GCC TAT GTT G
HOXA9	ATG AGA GCG GCG GAG ACA	CCA GTT GGC TGC TGG GTT A
HOXA10	AAA GCC TCG CCG GAG AA	GCC AGT TGG CTG CGT TTT
ERG	GAA CGA GCG CAG AGT TAT CGT	TGC CGC ACA TGG TCT GTA CT
LCOR	CTC AGT CAG AAC CTA GCG AAC AAG	GCC AGC ACA TGG ACT TTT CTT A
GAPDH	CCA CAT CGC TCA GAC ACC AT	GCG CCC AAT ACG ACC AAA T

**Table 4:** Conventional PCR primer sequences.

Human DNA	Gene Forward (5′–3′)	Reverse (5′–3′)
alpha-satellite, chromosome 17	GGGATAATTTCAGCTGACTAAACAG	TTCCGTTTAGTTAGGTGCAGTTATC
TRE3G-TF-FP	CTG GAG CAA TTC CAC AAC AC	
RUNX1c		CAA CGC CTC GCT CAT CTT
SPI1		GGA GCT CCG TGA AGT TGT TC
HOXA5		AGA TCC ATG CCA TTG TAG CC
HOXA9		CTT GGA CTG GAA GCT GCA C
HOXA10		CAG CTC TGC AGC CCG TAG
ERGv1		GTT CCT TGA GCC ATT CAC CT
LCOR		GGT CCA GAG GTG AGT CTT GG

**Table 5:** Antibody Details.

Antigen	Reactivity	Conjugated	Clone	Supplier
CD34	Human	APC-Cy7	581	BD Horizon Cat # 343514
CD31	Human	FITC		BD Horizon Cat # 555445
FLK1	Human	APC	89106	BD Horizon Cat # 560495
VE-cadherin	Human	PE-Cy7	16B1	BD Horizon Cat # 25-1449-41
CD45	Human	V450	2D1	BD Horizon Cat # 642275
CD43	Human	PE	1G10	BD Horizon Cat # 560199
CD235a	Human	PE	GA-R2	BD Horizon Cat # IM22114
CD45	Human	APC	2D1	BD Horizon Cat # 340943

**Table 6:** Gene expression sample details.

Lab Source	GEO ID	Symbol in Study	Samples Used	Sample IDs	Platform Technology	Total Annotated Genes
Bhatia	GSE3823	circle	18	U133A; GSM87705 to GSM87716, GSM87729 to GSM87734	HG U133A	13,462
Daley	GSE49938	diamond	17	GSM1210379 to GSM1210384, GSM1210388 to GSM1210392, GSM1210401 to GSM121406	HG U133A Plus2	23,520
Daley	GSE83719	triangle	5	All; GSM2214010 to GSM2299187	Illumina NextSeq 500	25,855

## Data Availability

Expression levels of RNA-seq and microarray data were obtained from series matrix sheets in the GEO repository (NCBI). (Table 6).

## Supplementary Materials

The following supporting information can be downloaded at: https://www.mdpi.com/article/10.3390/cells11121915/s1, Figure S1. Comparison of HE phenotype under normoxic or hypoxic conditions. Figure S2. Timeline of HE derivation using adapted protocol. Figure S3. Molecular validation of 7TF induction. Figure S4. Data from transplantation experiments.
