# Supplementary material for: Challenges in Cell Fate Acquisition to Scid-Repopulating Activity from Hemogenic Endothelium of hiPSCs Derived from AML Patients Using Forced Transcription Factor Expression
Source: Cells. 2022 Jun 13;11(12):1915. doi: 10.3390/cells11121915 (PMC9221973; doi:10.3390/cells11121915)
Supplement: Supplementary file 1 [file cells-11-01915-s001.zip › cells-1677287-supplementary.pdf]

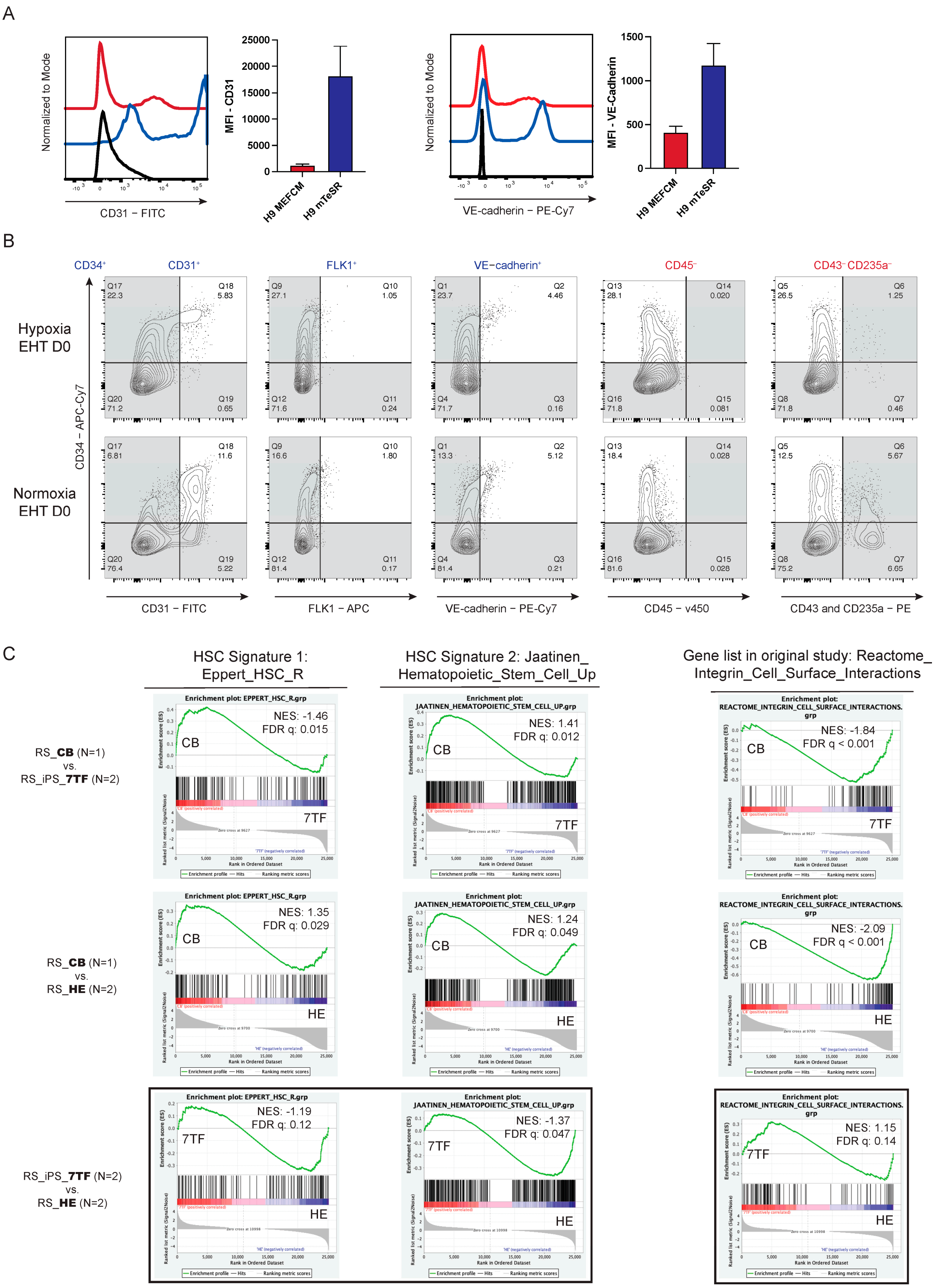

Supplemental Figure S1. Comparison of HE phenotype under normoxic or hypoxic conditions. (A) Histogram and corresponding bar graphs reporting flow cytometry analysis determined by MFI (mean fluorescence intensity) of CD31 or VE-Cadherin respectively. H9 MEFCM denotes HE derivation by using Wang et al., 2004 methodology. H9 mTeSR denotes derivation by using Sugimura et al., 2017 methodology. **(B)** Embryoid body differentiation in normoxia (5%CO_2) or hypoxia (5%CO_2/5%O_2/90% N_2) conditions. **(C)** Gene set enrichment analyses (GSEA) comparing gene expression profiles of the following samples from Sugimura et al.: CB, 7TF and HE. NES, normalized enrichment score; FDR, false discovery rate.

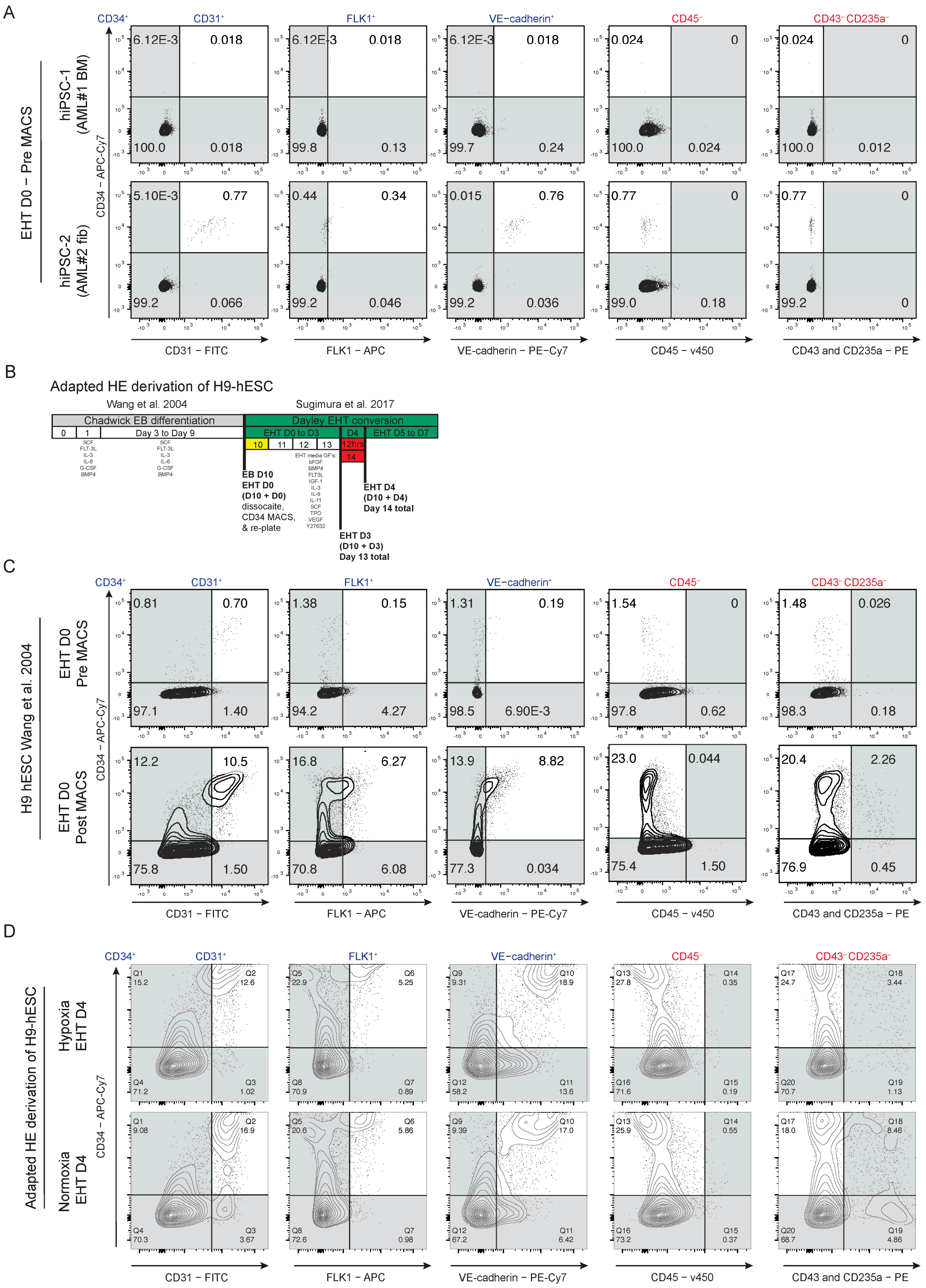

Supplemental Figure S2. Timeline of HE derivation using adapted protocol. (A) Flow analysis of HE phenotype on EHT day 0 pre CD34+ MACS enrichment in two AML-iPSC lines, hiPSC-1 (AML-iPSC derived from reprogramming AML patient 15331 bone marrow cells) and hiPSC-2 (AML-iPSC derived from reprogramming AML patient #2 fibroblast cells). **(B)** Schematic depicting timeline used for HE derivation by merging Wang et al., 2004 EB differentiation and Sugimura et al., 2017 endothelial-to-hematopoietic transition medium. **(C)** Flow analysis of HE phenotype on EHT day 0 pre and post CD34+ MACS enrichment of hESC (H9) using our adapted protocol. **(D)** Endothelial-to-hematopoietic transition differentiation in normoxic (5%CO_2) or hypoxic (5%CO_2/5%O_2/90% N_2) conditions at EHT day 4.

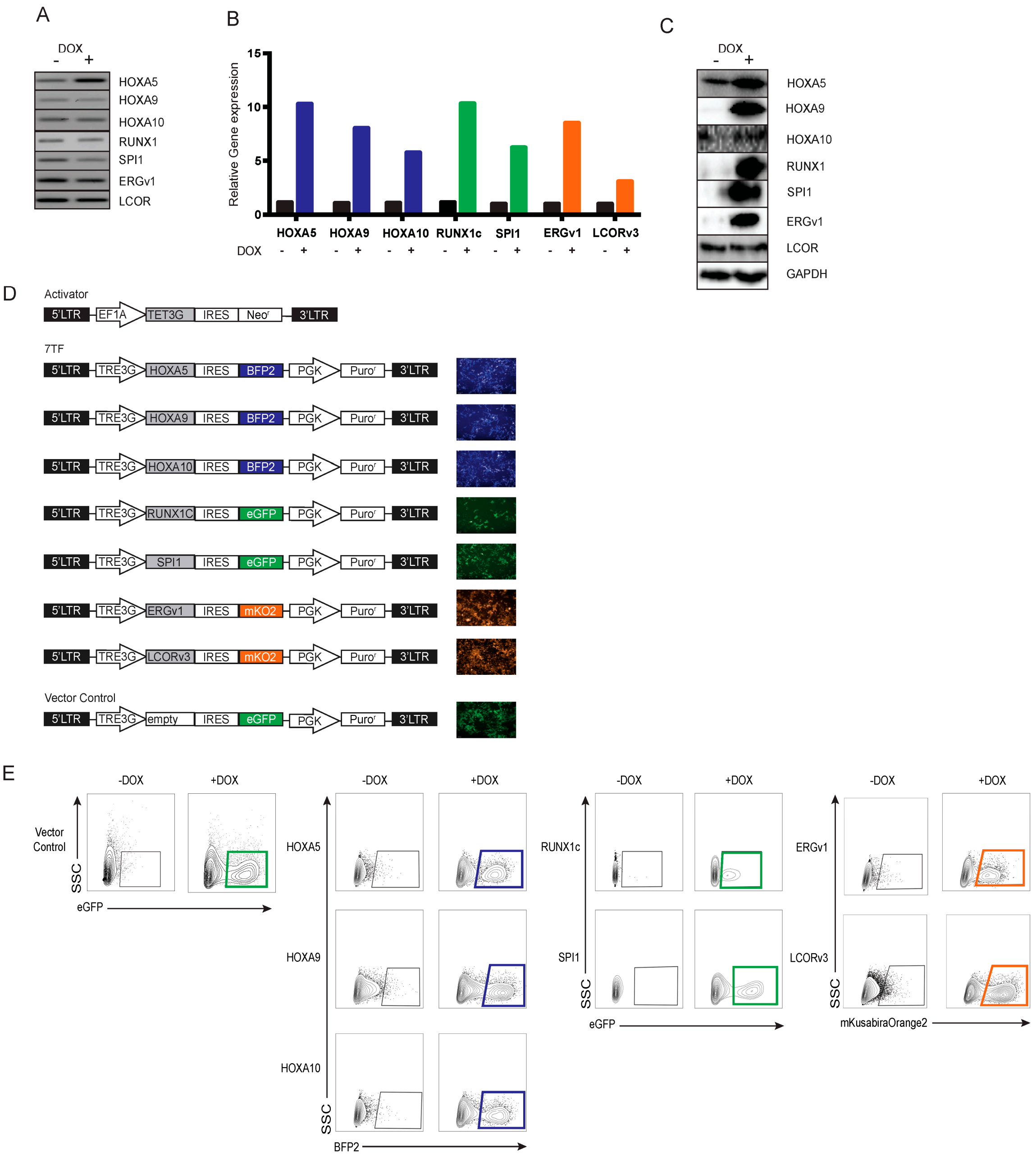

Supplemental Figure S3. Molecular validation of 7TF induction. (A) Validation of transcription factor by PCR (genomic integration) and **(B)** doxycycline induction of gene (qPCR) and **(C)** protein (WB) expression of TF on HEKs. **(D)** Vector constructs and representative fluorescent images of individual transcription factors acquired using the PerkinElmer Operetta High Content Imaging System. **(E)** Demonstration of fluorescent protein expression by flow cytometry on HEKs in the presence or absence of doxycycline.

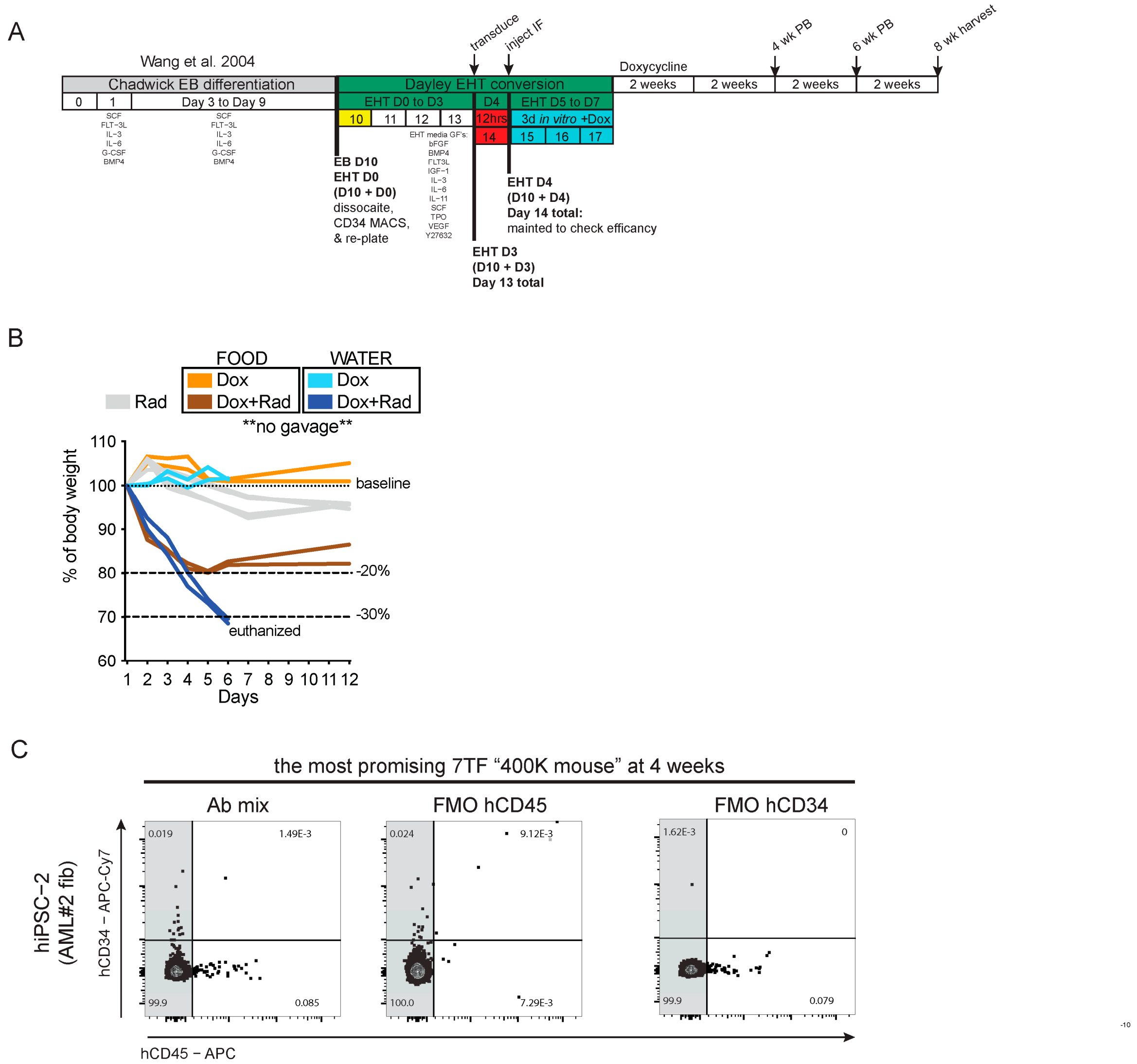

Supplemental Figure S4. Data from transplantation experiments. (A) Schematic depicting timeline of transplantation experiments utilizing adapted procedure for HE derivation by merging Wang et al., 2004 EB differentiation and Sugimura et al., 2017 endothelial-to-hematopoietic transition medium. **(B)** Observed issue resulting from combination of radiation and doxycycline (by food or water) leading to high morbidity rates. To mitigate this problem, 10g/L of sucrose was added to drinking water with doxycycline. Additionally, daily gavage was performed with dox & sucrose water, starting on the day of transplant. See methods for further details. **(C)** Representative flow plot of peripheral blood chimerism of "best" 400K mouse from hiPSC-2 = normal iPSC from AML #28787 fib clone N18
